# Vibration-Response-Only Structural Health Monitoring for Offshore Wind Turbine Jacket Foundations via Convolutional Neural Networks

**DOI:** 10.3390/s20123429

**Published:** 2020-06-17

**Authors:** Bryan Puruncajas, Yolanda Vidal, Christian Tutivén

**Affiliations:** 1 Control, Modeling, Identification and Applications (CoDAlab), Department of Mathematics, Escola d’Enginyeria de Barcelona Est (EEBE), Universitat Politècnica de Catalunya (UPC), Campus Diagonal-Besós (CDB), Eduard Maristany, 16, 08019 Barcelona, Spain; bpurunca@espol.edu.ec; 2 Mechatronics Engineering, Faculty of Mechanical Engineering and Production Science (FIMCP), Escuela Superior Politécnica del Litoral (ESPOL), Guayaquil 09-01-5863, Ecuador; cjtutive@espol.edu.ec

**Keywords:** structural health monitoring, damage detection, damage identification, offshore wind turbine foundation, jacket, signal-to-image conversion, convolutional neural network

## Abstract

This work deals with structural health monitoring for jacket-type foundations of offshore wind turbines. In particular, a vibration-response-only methodology is proposed based on accelerometer data and deep convolutional neural networks. The main contribution of this article is twofold: (i) a signal-to-image conversion of the accelerometer data into gray scale multichannel images with as many channels as the number of sensors in the condition monitoring system, and (ii) a data augmentation strategy to diminish the test set error of the deep convolutional neural network used to classify the images. The performance of the proposed method is analyzed using real measurements from a steel jacket-type offshore wind turbine laboratory experiment undergoing different damage scenarios. The results, with a classification accuracy over 99%, demonstrate that the stated methodology is promising to be utilized for damage detection and identification in jacket-type support structures.

## 1. Introduction

Globally, wind power generation capacity has increased exponentially since the early 1990s, and as of the end of 2019, this capacity amounted to 650 GW [1]. Whereas onshore wind turbines (WTs) have dominated new wind installations in the past, the growth of offshore WTs is poised to become the new leader because of steadier wind, in addition to vast regions where its installation is possible. In regard to the global offshore market, the cumulative installations have now reached 23 GW, representing 4% of total cumulative installations. Unfortunately, offshore WTs are placed in a harsh environment that originates from the wind and sea conditions [2]. As a consequence, offshore WTs require rigorous safety measures because it is extremely complicated to do operation and corrective work on these huge WTs placed in remote locations. Given that approaches centered on enhancing component reliability are likely to increase capital expenditures, system design optimization research and development activities should instead focus on minimizing and, if possible, even eliminating unexpected failures. In other words, the wind industry must abandon corrective maintenance (remedy failures) and move toward predictive maintenance (repair immediately before failure) to achieve maximum availability. Thus, the development of a structural health monitoring (SHM) strategy is particularly necessary to achieve this goal.

**Figure 1:**
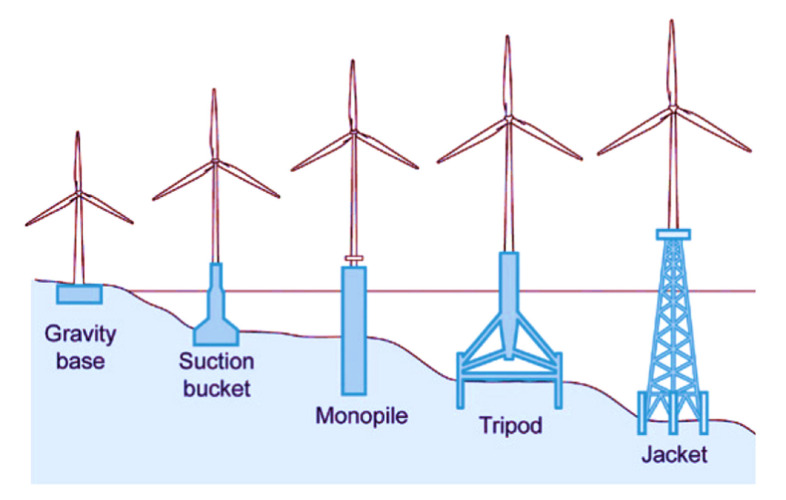
Fixed bottom wind turbine foundations [3].

Onshore and offshore fixed WTs differ mainly in the structure of their respective foundations. Several types of offshore foundations are used, with foundation choice depending on the water depth. The most common foundations are shown in Figure 1, see [3]. Note that jacket foundations, which are the object of study of this work, are preferred for greater depths (usually, between 30 to 90 m).

The detection of early-stage damage in the foundation is of great importance to avoid the possible future collapse of the entire structure. As stated in “Long-term research challenges in wind energy—a research agenda by the European Academy of Wind Energy” [4]:

A defining marine environment main characteristic is that structures are always subject to excitations. Techniques for structural health monitoring, vibration and data analysis must be capable of coping with such ambient excitations. As the input is typically not known, a normal input-output formalism cannot be used.

Thus, to overcome this challenge—which is posed by the fact that the input is typically not known—in this work, a structural health monitoring strategy for jacket-type foundations is developed based on a vibration response-only methodology. This is a challenge by itself as many of the works in the SHM field are based on the principle of guided elastic waves with a given (known) input excitation. See, for example, the overview of SHM systems for various WT components presented by Liu et al. [5]. In contrast, in this work, a new paradigm is introduced in which a predetermined excitation in the structure is no longer forced, but rather, the incident wind and waves serve as the exciting forces in the structure. In this way, the classic pattern recognition paradigm with identical excitation (e.g., [6]) becomes a problem of pattern recognition with variable excitation. Consequently, the new paradigm implies greater complexity in the damage detection process. Furthermore, until recently, few contributions have targeted offshore WT foundations. Notably, work by Weijtjens et al. [7] was focused on a real WT foundation and contributed an SHM strategy based on the resonance frequencies of the foundation. However, the results only proved some increased stiffness of the structure and could not give a final diagnosis about damage detection. Similarly, Oliveira et al. [8] introduced the main aspects in the development of a vibration-based monitoring system for an onshore 2.0-MW wind turbine based on identification of the modal properties of the most important vibration modes, in which detailed attention was given to the statistical procedure based on regression models that was used to minimize the influence of operational and environmental effects over the features considered to detect structural changes in the WT. However, only damage detection was pursued with a single type of damage. Noteworthily, the work by Zugasti [9] used damage estimators to detect damage in an experimental offshore tower similar to that employed in this work. Nevertheless, only damage detection was attained. In this work, in contrast to the aforementioned references, several types of damage are studied, and not only damage detection but also its classification is achieved.

It is important to note that the SHM standard approach for the problem at hand is usually an unsupervised one. That is, as no one would purposely damage their assets to train a SHM tool, only healthy data from the real structure is used. However, it is unfeasible to correctly identify different damage states using solely data obtained during what is assumed to be a healthy state. In this framework, detection can be accomplished by using a model of normality or unsupervised models, but not classification on the type of damage. The approach proposed in this work is the opposite, that is, a supervised approach. Thus, data from the damaged structure are required to train the model. In practice, this will be accomplished by means of computer models, as the finite element method (FEM). The FEM model should be validated with a downscaled experimental tower (as the one proposed in this work). Then, the full-scale finite element model would be used to generate healthy (to validate with the real asset) and damage samples. Finally, the stated supervised methodology proposed in this work can be used. In this work, a satisfactory experimental proof of concept has been conducted with the proposed strategy and a laboratory downscaled WT. However, future work is needed to validate the technology in a full-scale and more realistic environment. Some examples of this type of approach are given in [10], where bridge damage detection is accomplished by a neural network considering errors in baseline finite element models, and [11] where the stated SHM method for an oil offshore structure is capable to cope with several types of damage based on a finite element model.

On the one hand, it has been shown that traditional machine learning requires complex feature extraction processes and specialized knowledge, especially for a complex problem such as WT condition monitoring [12,13,14]. Moreover, extracting features with classic machine learning methods faces the classic bias-variance dilemma from inference theory. The bias–variance trade-off implies that a model should balance under-fitting and over-fitting; that is, the model should be rich enough to express underlying structure in the data but simple enough to avoid fitting spurious patterns, respectively. On the other hand, in the modern practice of deep learning, very rich models are trained to precisely fit (i.e., interpolate) the data. Classically, such models would be considered over-fit, and yet they often obtain high accuracy on test data. Thus, this paper proposes to use deep convolutional neural networks (CNN) for pattern recognition (classification), avoiding the aforementioned usual problems in the literature—e.g., [12,13,14]—related to feature extraction and bias–variance trade-off. In particular, we develop a novel damage diagnosis method for WT offshore foundations based on transforming condition monitoring multi-vibration-signals into images (with as many channels as sensors) to be processed afterward using deep CNN.

The paper is organized in the following manner. First, in Section 2, the experimental setup is introduced. It consists of a steel jacket-type offshore WT laboratory structure undergoing different damage scenarios. Then, in Section 3, the proposed SHM strategy is described in detail. The approach can be summarized by the following steps: (i) accelerometer data is gathered, (ii) a preprocess is designed to extract the maximum amount of information and to obtain a dataset of 24 (that is, the same number as accelerometer sensors) channel gray-scale images, (iii) 24-channel-input deep CNN is designed and trained for classification of the different structural states. In Section 4, the obtained results are conferred, showing an exceptional performance with all considered metrics giving results greater than 99%. Lastly, the main conclusions are given in Section 5 as well as future work research directions.

## 2. Experimental Setup

**Figure 2:**
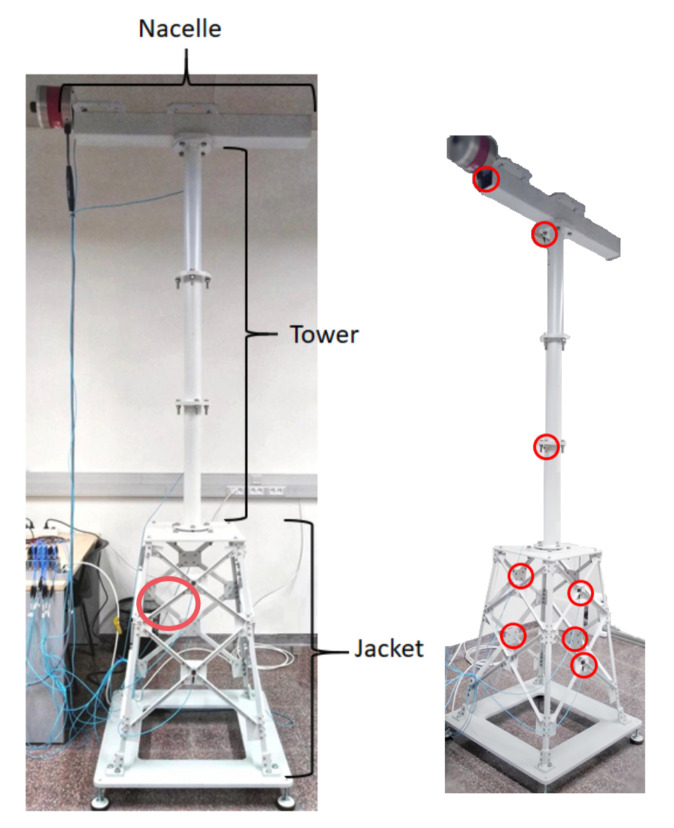
The experimental setup (**left**) detailing the location of the damaged bar (red circle). Location of the sensors on the overall structure (**right**).

The laboratory experimental setup is described in the following. First, a function generator (GW INSTEK AF-2005 model) is employed to generate a white noise signal. Then, this signal is amplified and applied to a modal shaker (GW-IV47 from Data Physics) that induces the vibration into the structure. The general overview of the experimental setup is shown in Figure 2 (left). The structure is 2.7 m tall and composed of three parts:

1. The top beam (1×0.6 m), where the modal shaker is attached to simulate a nacelle mass and the effects of wind excitation;
2. The tower with three tubular sections connected with bolts;
3. The jacket, which includes a pyramidal structure made up by 32 bars (S275JR steel) of different lengths, sheets (DC01 LFR steel), and other elements such as bolts and nuts.

It should be noted that different wind speeds are considered by modifying the white noise signal amplitude (i.e., scaling the amplitude by 0.5, 1, 2, and 3).

To measure vibration, eight triaxial accelerometers (PCB^®^ Piezotronic, model 356A17) are placed on the structure, see Figure 2 (right). The optimal number and placement of the sensors is determined according to [9]. The accelerometers are connected to six National Instruments™ cartridges (NI 9234 model) that are inserted into the National Instruments chassis cDAQ-9188. Finally, the Data Acquisition Toolbox™ is employed to configure the data acquisition hardware and read the data into MATLABmathsizesmall^®^.

**Figure 3:**
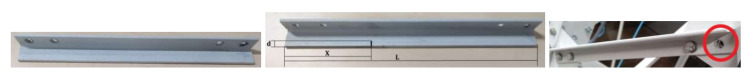
Different structural state scenarios studied in this work. Replica (healthy) bar (**left**). Crack damage, where *L* is the length of the bar, d= 5 mm is the crack size, and X=L/3 is the location of the crack in the bar (**center**). Missing bolt (**right**).

The studied damage states are related to one of the jacket bars, see Figure 3. The damage states include a 5-mm bar crack and loosening one of the jacket bolts. Furthermore, a pristine replica bar is also considered.

Finally, note that the purpose of the paper is to verify that the conceived methodology has practical potential. The laboratory tower is a simplified model, but it is valid for this preliminary study because it is similar to the laboratory towers used, for example, in [9], where damage detection is accomplished (but not localization or identification) via damage indicators; in [15,16], where statistical time series are employed to detect damage; and in [17,18], where damage detection is accomplished through principal component analysis and support vector machines.

## 3. Structural Health Monitoring Proposed Methodology

The proposed SHM strategy follows the steps detailed here. First, the raw time series data are collected. Second, the data are preprocessed to obtain a dataset of 24-channel gray-scale images. Third, a 24-channel-input CNN is designed and trained for classification of the different structural states. The following subsections describe the aforementioned procedure in detail.

### 3.1. Data Gathering

The data were gathered in different experiments with a sampling rate of 275.27 Hz and a duration of 60 s each. Table 1 shows the total number of realized experiments for the corresponding structural state (with its corresponding label) and white noise amplitude. A total of K=100 experiments were conducted. Given the *k*-th experiment, where *k* is varied from 1 to K=100, the raw data were then saved in the matrix $X^{\left(k\right)}\in M_{16517\times 24}\left(R\right)$

(1) $$\begin{array}{c} X^{\left(k\right)}=\left(\begin{array}{cccc} x_{1,1}^{\left(k\right)} & x_{1,2}^{\left(k\right)} & \cdots & x_{1,24}^{\left(k\right)}\\ x_{2,1}^{\left(k\right)} & x_{2,2}^{\left(k\right)} & \cdots & x_{2,24}^{\left(k\right)}\\ \vdots & \vdots & \ddots & \vdots \\ x_{16517,1}^{\left(k\right)} & x_{16517,2}^{\left(k\right)} & \cdots & x_{16517,24}^{\left(k\right)} \end{array}\right). \end{array}$$

Note that there are as many rows as the number of measurements in each experiment—that is, I=16,517—and as many columns as the number of sensors, J=24 (because each column is related to one sensor). Ultimately, the overall data matrix $X\in M_{1651700\times 24}\left(R\right)$ is constructed by stacking the matrices that arise from each different experiment,

(2) $$\begin{array}{c} X=\left(\begin{array}{c} X^{\left(1\right)}\\ \vdots \\ X^{\left(k\right)}\\ \vdots \\ X^{\left(100\right)} \end{array}\right). \end{array}$$

### 3.2. Data Preprocessing: Scaling, Reshaping, Augmentation, and Signal-To-Image Conversion

Data preprocessing is both the initial step and a critical step in machine learning. In this work, data reshaping is employed to guarantee that each sample includes multiple measurements from each sensor and thus has sufficient information to make a diagnosis regarding the state of the structure. Furthermore, a data-augmentation strategy is proposed to improve the final test set error of the prediction model. It is clear that the signal-to-image conversion as well as the architecture and hyperparameters of the deep CNN will play a key role in the damage detection methodology. However, the manner in which these data are scaled, augmented, and reshaped will significantly impact the overall performance of the strategy [19].

#### 3.2.1. Data Scaling

The importance of preprocessing techniques for image classification by CNN is well known [20]. The main reason for data scaling is to enhance the efficiency of the neural network training process, significantly decreasing the number of epochs required for the network to learn, and thus leading to a better predictor. In particular, here, the data are scaled column-wise to fall within the specific range [0,255]. This range is selected to later allow for easy conversion into gray-scale images. In particular, the range is computed as follows. Assuming that there are *K* experimental tests, *I* samples per experiment, and *J* sensors,

(3) $$\begin{array}{cc} M_{j} & =\max\left(x_{ij}^{\left(k\right)}\right),\;i=1,\ldots ,I,\;k=1,\ldots ,K, \end{array}$$

(4) $$\begin{array}{cc} m_{j} & =\min\left(x_{ij}^{\left(k\right)}\right),\;i=1,\ldots ,I,\;k=1,\ldots ,K, \end{array}$$

where $M_{j}$ and $m_{j}$ are the maximum and the minimum values, respectively, of all the measures at column *j*, where j=1,…,J. Accordingly, the elements of matrix X are scaled

(5) $$\begin{array}{cc} y_{ij}^{\left(k\right)} & :=\left(x_{ij}^{\left(k\right)}-m_{j}\right)\frac{255}{M_{j}-m_{j}},\;i=1,\ldots ,I,\;\;j=1,\ldots ,J,\;\;k=1,\ldots ,K, \end{array}$$

to create a new matrix Y as

(6) $$\begin{array}{c} Y=\left(\begin{array}{cccc} y_{1,1}^{\left(1\right)} & y_{1,2}^{\left(1\right)} & \cdots & y_{1,24}^{\left(1\right)}\\ \vdots & \vdots & \ddots & \vdots \\ y_{16517,1}^{\left(1\right)} & y_{16517,2}^{\left(1\right)} & \cdots & y_{16517,24}^{\left(1\right)}\\ \; & \;\\ \; & \;\\ y_{1,1}^{\left(2\right)} & y_{1,2}^{\left(2\right)} & \cdots & y_{1,24}^{\left(2\right)}\\ \vdots & \vdots & \ddots & \vdots \\ y_{16517,1}^{\left(2\right)} & y_{16517,2}^{\left(2\right)} & \cdots & y_{16517,24}^{\left(2\right)}\\ \; & \;\\ \; & \;\\ \vdots & \vdots & \ddots & \vdots \\ \; & \;\\ \; & \;\\ y_{1,1}^{\left(100\right)} & y_{1,2}^{\left(100\right)} & \cdots & y_{1,24}^{\left(100\right)}\\ \vdots & \vdots & \ddots & \vdots \\ y_{16517,1}^{\left(100\right)} & y_{16517,2}^{\left(100\right)} & \cdots & y_{16517,24}^{\left(100\right)} \end{array}\right)=\left(\begin{array}{cc} \; & \;\\ \; & Y^{\left(1\right)}\\ \; & \;\\ \; & \;\\ \; & \;\\ \; & \;\\ \; & Y^{\left(2\right)}\\ \; & \;\\ \; & \;\\ \; & \;\\ \; & \vdots \\ \; & \;\\ \; & \;\\ \; & \;\\ \; & Y^{\left(100\right)}\\ \; & \; \end{array}\right). \end{array}$$

#### 3.2.2. Data Reshaping

In this section, data reshaping is employed to guarantee that each sample has multiple measurements from each sensor and thus has sufficient information to diagnose the state of the structure. In particular, matrix (6) is reshaped to matrix $Z\in M_{\left(6400)\times (256\cdot 24\right)}$, as given in Table 2. It should be noted that the data in the first 256 columns are related to sensor 1 and define the first submatrix block, denoted as $Z_{1}$. Then, the data in columns 257 to 512 are related to sensor 2 and define the second submatrix block $Z_{2}$. Next, the columns 513 to 768 are related to sensor 3 and define the third submatrix block $Z_{3}$, and so on and so forth, until the last sensor related to $Z_{24}$ has been accounted for.

It should be noted that each row of matrix Z contains the information of one sample of our SHM strategy. Notice that to diagnose a WT, the trained model requires at least one sample. Based on the aforementioned reshaping process, the expected sample now contains 256 time stamps from each sensor. In this manner, less than 1 second is required to gather the necessary data when the sampling frequency is 275.27 Hz. Thus, this process leads to a faster detection time (amount of time that elapses between fault occurrence and detection). The intuition behind the proposed data reshape is twofold: (*i*) it supplies more information to each sample, and (ii) it simplifies the signal-to-image conversion, as stated in Section 3.2.4, because 256 is a perfect square.

Finally, observe that from matrices $Y^{\left(k\right)},\;k=1,\ldots ,K$ in Equation (6), the last samples $y_{i,j}^{\left(k\right)}$ from i=16385,⋯,16517, are discarded to reshape the data in the aforementioned new matrices $Z^{\left(k\right)},\;k=1,\ldots ,K$.

#### 3.2.3. Data Augmentation

Deep convolutional neural networks rely heavily on big data to avoid overfitting, see [21]. Unfortunately, many application domains lack access to big data. In this work, to build a better deep CNN model, a data augmentation strategy is proposed that artificially expands the size of the training dataset without actually collecting new data.

The method consists of using each time stamp as the beginning of a new sample (and using the subsequent 255 measures to complete the sample), as shown in Table 3. Accordingly, instead of the previously defined matrices (see Table 2) $Z^{\left(k\right)}\in M_{\left(64)\times (256\cdot 24\right)},\;k=1,\ldots ,K$, augmented matrices with the same number of columns but more rows are obtained, namely, $D^{\left(k\right)}\in M_{\left(16129)\times (256\cdot 24\right)},\;k=1,\ldots ,K$. Thus, from the initial 64 samples per experiment, we increased to 16,129 samples per experiment. This is an increment of 25,200% in the total number of samples in the dataset.

Finally, the data matrix $D\in M_{1612900\times (256\cdot 24)}\left(R\right)$—which contains the scaled, reshaped, and augmented data from all of the experiments—is defined by stacking the data matrices derived from each different experiment (recall that K=100),

(7) $$\begin{array}{c} D=\left(\begin{array}{c} D^{\left(1\right)}\\ \vdots \\ D^{\left(k\right)}\\ \vdots \\ D^{\left(100\right)} \end{array}\right). \end{array}$$

#### 3.2.4. Signal-To-Image Conversion

The fault diagnosis method converts time-domain signals from the 24 measured variables into 2D gray-scale images to exploit texture information from the converted images. The data conversion process was inspired based on reference [13], although the process is enhanced here by using multichannel images.

**Figure 4:**
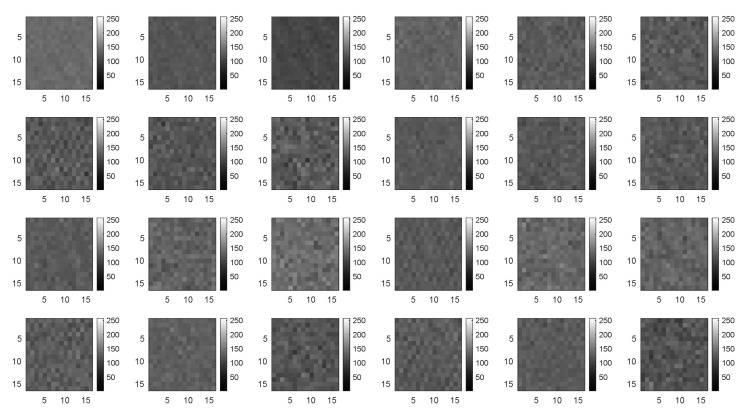
Multichannel gray-scale image corresponding to the 24 sensors (size 16×16).

The image size used for signal-to-image conversion is 16×16 (256 pixels) with 24 channels, constructed as follows. Each row of matrix D, see Equation (7), is converted to one image of size 16×16 with 24 channels (one channel per sensor), similar to a standard RGB image with 3 channels. It should be noted that because the sampling time is 1/257 seconds, each image contains approximately one second of data from each sensor, which is sufficient to capture all of the system dynamics. The total number of images in the dataset is 1,612,900, because 16,129 images are obtained from each of the 100 experiments. Figure 4 shows one example of such a multichannel image.

### 3.3. Deep Convolutional Neural Network

CNNs are feed-forward artificial neural networks that use the convolution operation instead of matrix multiplication. The preprocessing required in a CNN is significantly less than that required by other classification algorithms because features are not hand-engineered but learned. Typically, there are three kinds of layers: convolution, fully-connected, and softmax. The main aspects pf the convolution layer are its sparse local connectivity and filters, which significantly diminish the number of network parameters while simultaneously increasing its performance. The convolution layer’s last step is to apply the so-called activation function, which is a nonlinear function. Fully-connected layers are normal neural network layers in which all the outputs from the previous layer are connected to all the nodes in the next layer. Normally, these layers go towards the end of the network. Finally, a softmax layer assigns probabilities to each class and connects to the final output layer that will have the same number of neurons as classes.

To construct a deep CNN for a particular application is a complex task. In comparison to the wealth of research related to color images, very little work has been carried out for gray-scale images. In this work, a CNN is designed for the detection of different structural damage states based on 24-channel gray-scale images.

#### 3.3.1. Data Split: Training Set and Validation Set

To develop the classification model, deep learning methods divide the available data into training and validation sets. The training dataset is the actual dataset used to train the model (weights and biases in a CNN). In other words, the training dataset is the sample of data used to fit the model. In contrast, the validation dataset is the sample of data used to provide an unbiased evaluation of the model fit on the training dataset while tuning the model hyperparameters.

In this work, the following dataset split ratio has been used: 75% of the whole dataset is assigned to the training set, and 25% is assigned to the validation set. That is, 1,209,675 images with data augmentation, or 4800 without data augmentation, are used to train the CNN. Then, 403,225 images with data augmentation, or 1600 without data augmentation, are used to validate the model.

#### 3.3.2. Network Architecture

**Figure 5:**
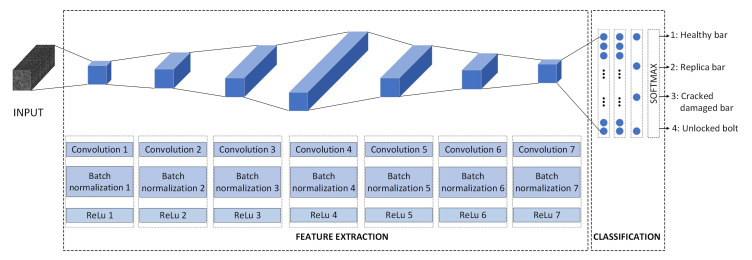
Architecture of the developed convolutional neural network (CNN).

The network presented in Figure 5 was designed in this work.

**Figure 6:**
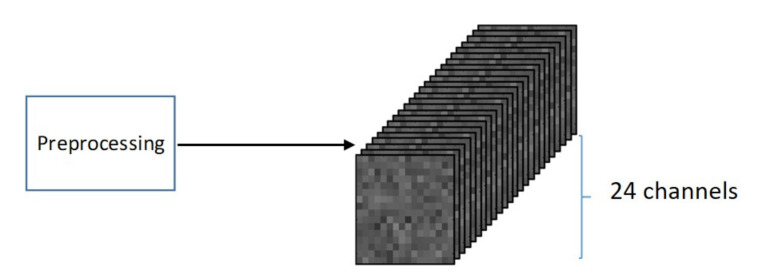
Example of one image in the dataset (24 channels) used as the CNN input.

The input is a 16×16 image with 24 channels, all of which are gray-scale. Figure 6 shows an example of one image in the dataset that was obtained after the preprocess procedure stated in Section 3.2.

The input is convoluted by a series of 7 convolutional layers. Each convolution layer is followed by a batch normalization step, which is used to improve the speed, performance, and stability of the CNN [22]; and a ReLU (Rectified Linear Unit) activation function (f(x)=max(0,x)), because this approach has been shown to speed up the training process in comparison to the classic sigmoid alternative. The final layers of the network are three fully connected layers and a softmax block, used to squash the 4-dimensional output into a categorical probability distribution: (1) original healthy bar, (2) replica bar, (3) crack damaged bar, and (4) unlocked bolt.

The most significant characteristics of the CNN architecture are summarized in Table 4.

It should be noted that the convolutions with a maximum number of parameters are the intermediate case (convolutions 4 and 5), whereas those with the minimum number of parameters correspond to the first and last convolutions. Finally, the three fully connected layers have sizes 32, 16, and 4, respectively, and are followed by the softmax function with four outputs.

It should also be noted that each convolution employs a padding of 1. The main intuition behind this selection is that normally, the filter is applied by superimposing it on the image from the upper left edge. Then, a columnar translation is applied until the filter is superimposed with its right edge on the right edge of the image. This usual way of proceeding has a problem, the edge pixels are never subjected to the central part of the filter. This is sometimes known as the border effect problem and can be solved by incorporating so-called padding [23]. That is to apply the filter beginning from outside the image frame as well as ending also outside the image, in such a manner that edge pixels reach also the center part of the filter. In this work, a padding of 1 is used to enhance the texture features extracted by the CNN for all of the data in the image, regardless of whether the data are located in the image. Table 5 compares different metrics (see Section 4.1, where a definition of these metrics is given) with and without padding (without data augmentation). It can be observed that when using padding, better results are attained.

#### 3.3.3. Network Training

The training of the CNN consists of the minimization of a loss function by means of a numerical optimization algorithm. In this work, the Adam optimizer [24] is employed to minimize the categorical cross entropy [25]. The Adam algorithm combines two versions of speeding up gradient descent: (i) gradient descent with momentum, where the basic idea is to compute an exponentially weighted average of the gradients, and (ii) root mean square propagation (RMSProp), that makes use of the gradient second moments. Specifically, the Adam numerical method puts together the exponential moving average of the gradient and the squared gradient (second moment), and hyperparameters $\beta_{1}$ and $\beta_{2}$ handle their decrease rates, respectively. In this work, the Adam optimizer has been tuned and thus employs an initial learning rate of $\alpha_{0}=0.01$, and values $\beta_{1}=0.9$, $\beta_{2}=0.992$, and $\varepsilon =10^{-7}$ to avoid divisions by zero. Furthermore, here, the learning rate is decreased every 2 epochs by multiplying with factor 0.5.

Convolutional layer initialization is carried out by the so-called Xavier initializer [26]. Mini-batches of size 75 in the initial dataset and 590 for the augmented dataset are used to update the weights.

Finally, L2 regularization with $\lambda =10^{-6}$ is employed. Table 5 compares the different metrics (see Section 4.1 for a definition of these metrics) with and without L2 regularization (without data augmentation). It can be observed that when using regularization, better results are obtained because regularization reduces high variance in the validation set.

#### 3.3.4. Network Architecture and Hyperparameter Tuning

To select the best architecture and to tune the different hyperparameters usually requires significant computational resources. As one of the most critical aspects of computational cost is the dataset size, in this paper, following the results presented in [27,28], the small dataset (without augmentation) is used to define the CNN architecture and quickly (coarse) tune the hyperparameters. Next, the obtained optimal hyperparameters for the small dataset are used as initial values to fine-tune the hyperparameters with the large dataset (with data augmentation).

#### 3.3.5. Network Implementation

The stated methodology is coded in MATLABmathsizesmall^®^ using its Deep Learning Toolbox™ on a laptop running the Windows^®^ 10 operating system, with an Intel Core i7-9750H processor, 16 GB of RAM, and an Nvidia GeForce RTX™2060 graphics card that has 6 GB of dedicated VRAM.

## 4. Results and Discussion

### 4.1. Metrics to Evaluate the Classification Model

To measure classification performance, several metrics can be computed from a confusion matrix such as that shown in Table 6. Normally, these metrics evaluate binary classification problems. Note that true positive (TP) is the number of positive samples that are correctly predicted as such, false positive (FP) is the number of negative samples that are incorrectly predicted, true negative (TN) is the number of negative samples that are correctly predicted, and false negative (FN) is the number of positive samples that are incorrectly predicted.

The most common metrics for binary classification problems are the following.

- Accuracy: proportion of true results (both true positives and true negatives) among the total number of cases examined.
  $$\mathrm{Accuracy}=\frac{\mathrm{TP}\;+\;\mathrm{TN}}{\mathrm{TP}\;+\;\mathrm{FP}\;+\;\mathrm{FN}\;+\;\mathrm{TN}}$$
- Precision: proportion of positive results that are true positive.
  $$\mathrm{Precision}=\frac{\mathrm{TP}}{\mathrm{TP}\;+\;\mathrm{FP}}$$
- Recall: proportion of actual positives that are correctly identified as such.
  $$\mathrm{Recall}=\frac{\mathrm{TP}}{\mathrm{TP}\;+\;\mathrm{FN}}$$
- Specificity: proportion of actual negatives that are correctly identified as such.
  $$\mathrm{Specificity}=\frac{\mathrm{TN}}{\mathrm{TN}\;+\;\mathrm{FP}}$$
- F1-score: harmonic mean of the precision and recall.
  $$F1=2\cdot \frac{\mathrm{Precision}\;\cdot \;\mathrm{Recall}}{\mathrm{Precision}\;+\;\mathrm{Recall}}$$

In a multiclass classification problem, such as that considered in this work, these metrics are also applicable using a one-vs.-all approach to compute each metric for each class, see [29]. This is, essentially, to compute the different metrics for each label as if the problem has been reduced to a binary ’label *X*’ versus ’not label *X*’ situation.

### 4.2. Results of the CNN Classification Method

**Figure 7:**
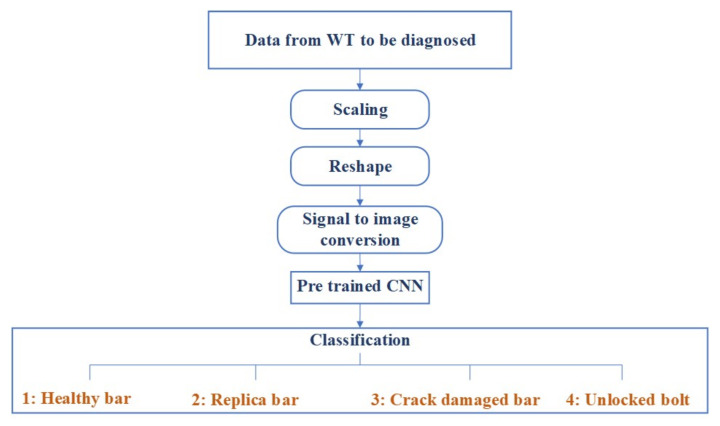
Flowchart to illustrate how the proposed structural health monitoring (SHM) strategy is applied when a wind turbine (WT) must be diagnosed.

To evaluate the developed methodology, this section presents the results obtained from the proposed SHM strategy. A flowchart of the proposed approach is given in Figure 7. When a WT must be diagnosed, the accelerometer data are scaled, reshaped, and converted into gray-scale images that are fed into the already trained CNN, and a classification is obtained to predict the structural state condition.

To thoroughly test the functional characteristics of the algorithm, the datasets with and without data augmentation are considered, as well as comparison with two other methodologies given in [9,17] that make use of the same laboratory structure. The first methodology, given in [17], is based on principal component analysis and support vector machines. The second methodology, given in [9] (page 67), is based on the well-known damage indicators: covariance matrix estimate and scalar covariance.

**Figure 8:**
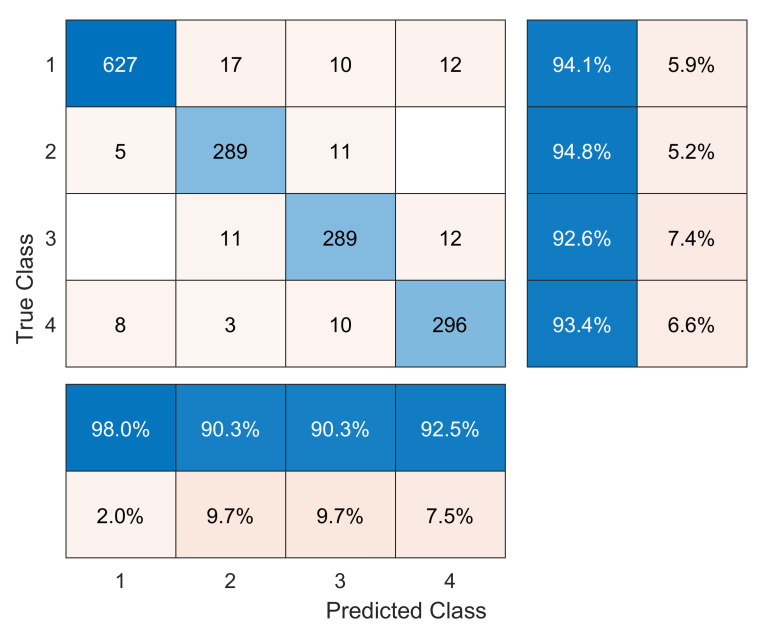
Confusion matrix for the validation dataset without data augmentation.

**Figure 9:**
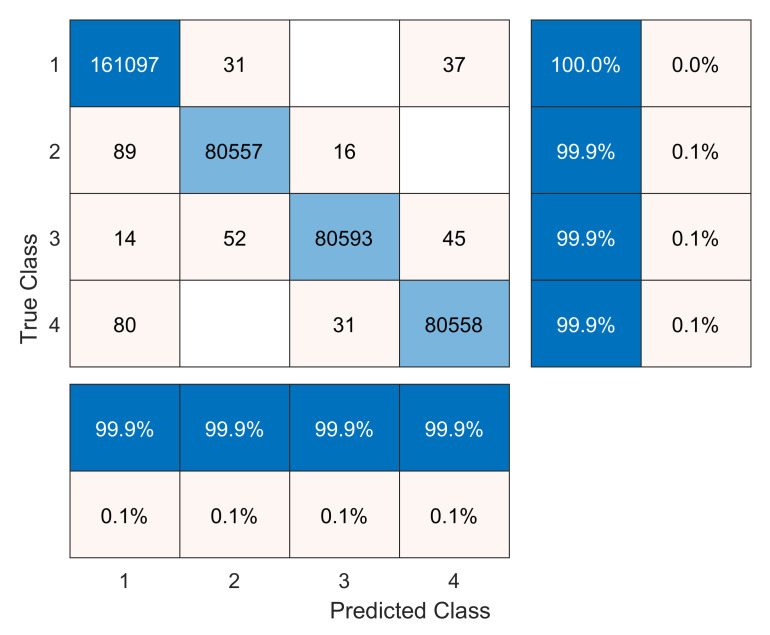
Confusion matrix for the validation dataset with data augmentation.

Figure 8 and Figure 9 illustrate the confusion matrices for the validation dataset without and with data augmentation, respectively. The rows represent the true class, whereas columns represent the predicted class. The precision and false discovery rate are given in the rightmost columns. Finally, the recall and false negative rate are given at the bottom rows. An examination of both confusion matrices reveals that some misclassifications come from the model confounding the healthy and replica bars (labels 1 and 2). However, this level of misclassification is acceptable because both bars are in a healthy state. In contrast, some errors are derived from the model misclassifying the crack and unlocked bolt damages (labels 3 and 4), which will not correctly detect the type of damage but would at least lead to a damage alert. Finally, it should be noted that very few damaged samples (labels 3 and 4) are classified as healthy or replica bar (labels 1 and 2).

From the confusion matrices, the different metrics to evaluate the classification model, see Section 4.1, are computed and presented in Table 7.

The impact of the data augmentation strategy can clearly be seen. Although no new experimental data were collected, nonetheless the metrics were significantly improved. It should be noted that all of the metrics (precision, recall, F1-score, and specificity) are higher than or equal to 99.86% for each label when using the augmented dataset in comparison to values between 90.31% and 98.61% for the initial dataset. Despite all metrics being relevant, considering the specific problem at hand, the most important metric is recall, which is the proportion of actual damaged cases that are correctly identified as such. It can be observed that the crack damage and the unlocked bolt, even without data augmentation, obtain recall values of 92.63% and 93.38%, respectively. When data augmentation is used, the recall values are all higher than or equal to 99.86% for all of the studied classes. The results associated with the precision metric are also satisfactory. When the initial dataset is used, precision values are between 90.31 and 97.97, but with the augmented dataset, such values are all higher than or equal to 99.89. Finally, it should be noted that the specificity metric is that which experiences less improvement when using the augmented dataset.

As already mentioned before, here, a comparison is made between our obtained results and two other methodologies. On the one hand, when using the first approach stated in [17], the crack damaged bar has a recall of 96.08%, and is thus inferior to the one obtained with the proposed strategy in this work which attained a value of 99.86%. Note that the crack damage is the most challenging. In fact, the second approach stated in [9] (page 82) was not capable of detecting this type of incipient damage when using the scalar covariance or mean residual damage indicators. On the other hand, the first approach obtains a recall of 99.02% for the unlocked bold damage; whereas with the proposed strategy, a slightly higher value of 99.86% is obtained. Finally, note that the unlocked bold damage is not studied in the second approach.

The proposed CNN exhibits low bias and variance for both datasets, because the training and validation errors are small (low bias), as well as the difference between them (low variance), as shown in Table 8. In particular, when using the initial dataset, the training error is equal to 0.1167 and the validation error is quite close to this same value, being equal to 0.1692. When using the augmented dataset, the training error diminishes to 0.0026 and the validation error is only slightly greater at 0.0044. From this table, the significantly increased training time (1196 min) of the augmented dataset in comparison to that of the initial dataset (11 min) can be seen, which is easily understood due to the size of each dataset. That is, there are 1,612,800 images in the augmented dataset and only 6400 images in the initial.

**Figure 10:**
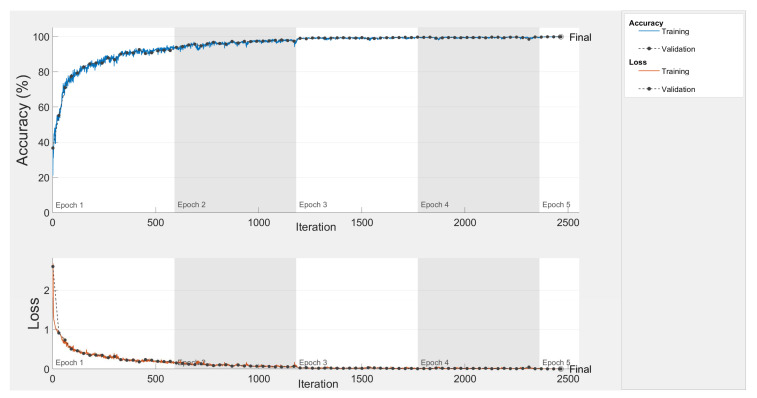
Accuracy and loss curve for the augmented dataset.

Finally, Figure 10 shows the accuracy and loss curves during training and validation (black dotted lines) when using the augmented dataset. It should be noted that after 5 epochs, the CNN obtains an accuracy of 99.90% and a final validation loss of 0.0044, as shown in Table 8.

## 5. Conclusions and Future Work

In this work, a strategy based solely on vibration response was demonstrated for the structural health monitoring of offshore WT foundations. The approach was tested on a laboratory setup, for which four different structural states for a jacket bar were studied: healthy bar, replica, crack damage, and an unlocked bolt.

The contribution of this work is twofold: (i) how three-dimensional data (derived from different time, sensors, and experiments) are preprocessed (collected, scaled, reshaped, augmented, and converted into gray-scale images with as many channels as sensors), and (ii) the design of a deep CNN, the architecture and hyperparameters of which play a key role in the specific application that concerns us—damage diagnosis. Furthermore, the proposed method does not require hand-designed features beforehand because the CNN learns features automatically.

The conceived SHM methodology with data augmentation shows exceptional performance, with all considered metrics (accuracy, precision, recall, F1-score, and specificity) giving results greater than 99.8%. In particular, a noteworthy overall accuracy of 99.90% is obtained with data augmentation. These results show that large (deep) CNNs are promising for the development of SHM strategies for WT offshore foundations.

Future work will focus on three main areas. First, based on open set domain adaptation [30], research to render capability of separating unknown damage from known targeted types of damage will be conducted. Second, not only detection and classification but also the localization of the damage will be attempted by designing an ensemble of deep CNNs, the main idea being to take advantage of individual information from each sensor signal. Lastly, to deal with the validation of the proposed strategy in a more realistic environment, a water tank facility will be used, in which the laboratory tower will be placed and subjected to the action of regular and irregular waves.

## Figures and Tables

**Table 1:** Total number of experimental tests for the different white noise (WN) amplitudes and for each structural state.

Label	Structural State	0.5 WN	1 WN	2 WN	3 WN
1	Healthy bar	10 tests	10 tests	10 tests	10 tests
2	Replica bar	5 tests	5 tests	5 tests	5 tests
3	Crack damaged bar	5 tests	5 tests	5 tests	5 tests
4	Unlocked bolt	5 tests	5 tests	5 tests	5 tests

**Table 2:** Data reshaping. On the one hand, this process can be viewed as the vertical stacking of K=100 matrices $Z^{\left(k\right)},\;k=1,\ldots ,K$, where each matrix is associated with a different experiment. On the other hand, this process can also be viewed as the horizontal concatenation of J=24 matrices, $Z_{j},\;j=1,\ldots ,J$, where each matrix is associated with a different sensor.

Sensor 1	…	Sensor 24
Z = $\left(\begin{array}{ccc} y_{1,1}^{\left(1\right)} & \cdots & y_{256,1}^{\left(1\right)}\\ y_{257,1}^{\left(1\right)} & \cdots & y_{512,1}^{\left(1\right)}\\ \vdots & \ddots & \vdots \\ y_{16129,1}^{\left(1\right)} & \cdots & y_{16384,1}^{\left(1\right)}\\ \vdots & \ddots & \vdots \\ y_{1,1}^{\left(k\right)} & \cdots & y_{256,1}^{\left(k\right)}\\ y_{257,1}^{\left(k\right)} & \cdots & y_{512,1}^{\left(k\right)}\\ \vdots & \ddots & \vdots \\ y_{16129,1}^{\left(k\right)} & \cdots & y_{16384,1}^{\left(k\right)}\\ \vdots & \ddots & \vdots \\ y_{1,1}^{\left(100\right)} & \cdots & y_{256,1}^{\left(100\right)}\\ y_{257,1}^{\left(100\right)} & \cdots & y_{512,1}^{\left(100\right)}\\ \vdots & \ddots & \vdots \\ y_{16129,1}^{\left(100\right)} & \cdots & y_{16384,1}^{\left(100\right)} \end{array}\right.$	⋯	$\left.\begin{array}{ccc} y_{1,24}^{\left(1\right)} & \cdots & y_{256,24}^{\left(1\right)}\\ y_{257,24}^{\left(1\right)} & \cdots & y_{512,24}^{\left(1\right)}\\ \vdots & \ddots & \vdots \\ y_{16129,24}^{\left(1\right)} & \cdots & y_{16384,24}^{\left(1\right)}\\ \vdots & \ddots & \vdots \\ y_{1,24}^{\left(k\right)} & \cdots & y_{256,24}^{\left(k\right)}\\ y_{257,24}^{\left(k\right)} & \cdots & y_{512,24}^{\left(k\right)}\\ \vdots & \ddots & \vdots \\ y_{16129,24}^{\left(k\right)} & \cdots & y_{16384,24}^{\left(k\right)}\\ \vdots & \ddots & \vdots \\ y_{1,24}^{\left(100\right)} & \cdots & y_{256,24}^{\left(100\right)}\\ y_{257,24}^{\left(100\right)} & \cdots & y_{512,24}^{\left(100\right)}\\ \vdots & \ddots & \vdots \\ y_{16129,24}^{\left(100\right)} & \cdots & y_{16384,24}^{\left(100\right)} \end{array}\right)=$$\left(\begin{array}{c} Z^{\left(1\right)}\\ \vdots \\ Z^{\left(k\right)}\\ \vdots \\ Z^{\left(100\right)} \end{array}\right)$=$\left(\begin{array}{ccc} Z_{1} & \left|\;\cdots \;\right| & Z_{24} \end{array}\right)$

**Table 3:** Synthetic data augmentation for experiment k,k=1,…,K.

	Signal 1	Signal 2	…	Signal 24
$D^{\left(k\right)}$ =	$\left(\begin{array}{ccc} y_{1,1}^{\left(k\right)} & \cdots & y_{256,1}^{\left(k\right)}\\ y_{2,1}^{\left(k\right)} & \cdots & y_{257,1}^{\left(k\right)}\\ y_{3,1}^{\left(k\right)} & \cdots & y_{258,1}^{\left(k\right)}\\ y_{4,1}^{\left(k\right)} & \cdots & y_{259,1}^{\left(k\right)}\\ \vdots & \ddots & \vdots \\ y_{16129,1}^{\left(k\right)} & \cdots & y_{16384,1}^{\left(k\right)} \end{array}\right.$	$\begin{array}{ccc} y_{1,2}^{\left(k\right)} & \cdots & y_{256,2}^{\left(k\right)}\\ y_{2,2}^{\left(k\right)} & \cdots & y_{257,2}^{\left(k\right)}\\ y_{3,2}^{\left(k\right)} & \cdots & y_{258,2}^{\left(k\right)}\\ y_{4,2}^{\left(k\right)} & \cdots & y_{259,2}^{\left(k\right)}\\ \vdots & \ddots & \vdots \\ y_{16129,2}^{\left(k\right)} & \cdots & y_{16384,2}^{\left(k\right)} \end{array}$	$\begin{array}{c} \cdots \end{array}$	$\left.\begin{array}{ccc} y_{1,24}^{\left(k\right)} & \cdots & y_{256,24}^{\left(k\right)}\\ y_{2,24}^{\left(k\right)} & \cdots & y_{257,24}^{\left(k\right)}\\ y_{3,24}^{\left(k\right)} & \cdots & y_{258,24}^{\left(k\right)}\\ y_{4,24}^{\left(k\right)} & \cdots & y_{259,24}^{\left(k\right)}\\ \vdots & \ddots & \vdots \\ y_{16129,24}^{\left(k\right)} & \cdots & y_{16384,24}^{\left(k\right)} \end{array}\right)$

**Table 4:** Characteristics of the designed CNN. The neural network has a total of 2,176,308 parameters. The number of output channels in each layer is highlighted in boldface font.

Layer	Ouput Size	Parameters	# of Parameters
Input 16 × 16 × 24 images	16 × 16 × **24**	-	0
Convolution#1 32 filters of size 5 × 5 × 24 with padding [1 1 1 1]	14 × 14 × **32**	Weight 5 × 5 × 24 × 32 Bias 1 × 1 × 32	19,232
Batch Normalization#1	14 × 14 × **32**	Offset 1 × 1 × 32 Scale 1 × 1 × 32	64
ReLu#1	14 × 14 × **32**	-	0
Convolution#2 64 filters of size 5 × 5 × 24 with padding [1 1 1 1]	12 × 12 × **64**	Weight 5 × 5 × 32 × 64 Bias 1 × 1 × 64	51,264
Batch Normalization#2	12 × 12 × **64**	Offset 1 × 1 × 64 Scale 1 × 1 × 64	128
ReLu#2	12 × 12 × **64**	-	0
Convolution#3 128 filters of size 5 × 5 × 24 with padding [1 1 1 1]	10 × 10 × **128**	Weight 5 × 5 × 64 × 128 Bias 1 × 1 × 128	204,928
Batch Normalization#3	10 × 10 × **128**	Offset 1 × 1 × 128 Scale 1 × 1 × 128	256
ReLu#3	10 × 10 × **128**	-	0
Convolution#4 256 filters of size 5 × 5 × 24 with padding [1 1 1 1]	8 × 8 × **256**	Weight 5 × 5 × 128 × 256 Bias 1 × 1 × 256	819,456
Batch Normalization#4	8 × 8 × **256**	Offset 1 × 1 × 256 Scale 1 × 1 × 256	512
ReLu#4	8 × 8 × **256**	-	0
Convolution#5 128 filters of size 5 × 5 × 24 with padding [1 1 1 1]	6 × 6 × **128**	Weight 5 × 5 × 256 × 128 Bias 1 × 1 × 128	819,456
Batch Normalization#5	6 × 6 × **128**	Offset 1 × 1 × 128 Scale 1 × 1 × 128	256
ReLu#5	6 × 6 × **128**	-	0
Convolution#6 64 filters of size 5 × 5 × 24 with padding [1 1 1 1]	4 × 4 × **64**	Weight 5 × 5 × 128 × 64 Bias 1 × 1 × 64	204,864
Batch Normalization#6	4 × 4 × **64**	Offset 1 × 1 × 64 Scale 1 × 1 × 64	128
ReLu#6	4 × 4 × **64**	-	0
Convolution#7 32 filters of size 5 × 5 × 24 with padding [1 1 1 1]	2 × 2 × **32**	Weight 5 × 5 × 64 × 32 Bias 1 × 1 × 32	51,232
Batch Normalization#7	2 × 2 × **32**	Offset 1 × 1 × 32 Scale 1 × 1 × 32	64
ReLu#7	2 × 2 × **32**	-	0
Fully connected layer#1	1 × 1 × **32**	Weight 32 × 128 Bias 32 × 1	4128
Fully connected layer#2	1 × 1 × **16**	Weight 16 × 32 Bias 16 × 1	528
Fully connected layer#3	1 × 1 × **4**	Weight 4 × 16 Bias 4 × 1	68
Softmax	-	-	0
classoutput	-	-	0

**Table 5:** Metrics for different CNN architectures without data augmentation. The best metric results are highlighted in boldface font.

Strategy	Accuracy	Precision	Recall	F1 Score	Specificity
ReLu-Padding-L2 regularization	**93.81**	**92.77**	**93.73**	**93.22**	**97.98**
Relu-No padding-L2 regularization	93.69	92.73	93.44	93.07	97.92
Relu-Padding-No L2 regularization	93.63	92.73	93.82	93.25	97.89

**Table 6:** Binary confusion matrix.

		Predicted Class	
		Positive	Negative
**Actual class**	**Positive**	True positive (TP)	False negative (FN)
	**Negative**	False positive (FP)	True negative (TN)

**Table 7:** Metrics for each label of the multiclassification problem and comparison between the datasets without and with data augmentation.

Dataset	Label	Precision	Recall	F1-Score	Specificity
Without data augmentation	1: Healthy bar	97.97	94.14	96.02	98.61
	2: Replica bar	90.31	94.75	92.48	97.61
	3: Crack damaged bar	90.31	92.63	91.46	97.59
	4: Unlocked bolt	92.50	93.38	92.94	98.13
With data augmentation	1: Healthy bar	99.89	99.96	99.92	99.92
	2: Replica bar	99.90	99.87	99.88	99.97
	3: Crack damaged bar	99.94	99.86	99.90	99.99
	4: Unlocked bolt	99.90	99.86	99.88	99.97

**Table 8:** Comparison of obtained accuracy, validation error, training error, and training time when using data augmentation with respect to the original dataset.

	Accuracy	Validation Error	Training Error	Training Time	# of Images
Whitout data augmentation	93.81	0.1692	0.1167	11 min	6400
With data augmentation	99.90	0.0044	0.0026	1196 min	1,612,800
